# *N*-Benzyl-9-isopropyl-9*H*-purin-6-amine

**DOI:** 10.1107/S1600536813013500

**Published:** 2013-05-25

**Authors:** David Gergela, Michal Rouchal, Peter Bartoš, Robert Vícha

**Affiliations:** a Department of Chemistry, Faculty of Technology, Tomas Bata University in Zlin, Nám. T. G. Masaryka 275, Zlín 762 72, Czech Republic; b Department of Chemistry, Faculty of Science, Masaryk University, Kamenice 5, Brno-Bohunice 625 00, Czech Republic

## Abstract

The asymmetric unit of the title compound, C_15_H_17_N_5_, consists of two mol­ecules in which the dihedral angles between the best planes of the purine ring system (r.m.s. deviations = 0.0060 and 0.0190 Å) and the benzene ring are 89.21 (3) and 82.14 (4)°. The mol­ecules within the asymmetric unit are linked into dimers by pairs of N—H⋯N hydrogen bonds. Weak C—H⋯π contacts and π–π inter­actions [centroid–centroid = 3.3071 (1) Å] further connect the mol­ecules into a three-dimensional network.

## Related literature

The title compound was prepared according to a modified procedure published by Fiorini & Abel (1998 ▶). For the biological activity of 6,9-disubstituted purines, see: Cappellacci *et al.* (2011 ▶); Jorda *et al.* (2011 ▶); Tunçbilek *et al.* (2009 ▶). For crystallographic data for related compounds, see: Novotná & Trávníček (2013 ▶); Rouchal *et al.* (2009*a* ▶,*b* ▶); Trávníček *et al.* (2010 ▶).
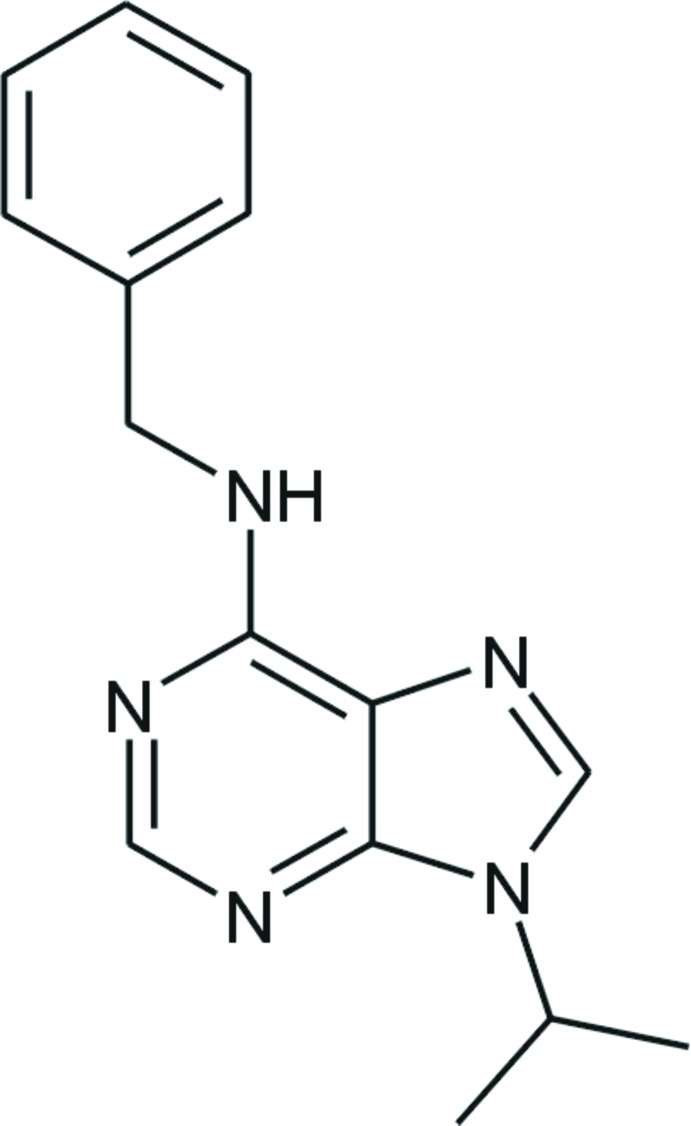


## Experimental

### 

#### Crystal data

C_15_H_17_N_5_
*M* *_r_* = 267.34
Monoclinic, 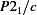

*a* = 12.9926 (5) Å
*b* = 21.1673 (7) Å
*c* = 11.2622 (6) Å
β = 114.274 (5)°
*V* = 2823.5 (2) Å^3^
*Z* = 8
Mo *K*α radiation
μ = 0.08 mm^−1^
*T* = 120 K
0.50 × 0.38 × 0.20 mm

#### Data collection

Oxford Diffraction Xcalibur (Sapphire2) diffractometer
Absorption correction: multi-scan (*CrysAlis RED*; Oxford Diffraction, 2009 ▶) *T*_min_ = 0.942, *T*_max_ = 1.000
21460 measured reflections
4972 independent reflections
3280 reflections with *I* > 2σ(*I*)
*R*_int_ = 0.035

#### Refinement

*R*[*F* ^2^ > 2σ(*F* ^2^)] = 0.029
*wR*(*F* ^2^) = 0.058
*S* = 0.83
4972 reflections
373 parameters
H atoms treated by a mixture of independent and constrained refinement
Δρ_max_ = 0.13 e Å^−3^
Δρ_min_ = −0.16 e Å^−3^

### 

Data collection: *CrysAlis CCD* (Oxford Diffraction, 2009 ▶); cell refinement: *CrysAlis RED* (Oxford Diffraction, 2009 ▶); data reduction: *CrysAlis RED*; program(s) used to solve structure: *SHELXS97* (Sheldrick, 2008 ▶); program(s) used to refine structure: *SHELXL97* (Sheldrick, 2008 ▶); molecular graphics: *ORTEP-3 for Windows* (Farrugia, 2012 ▶) and *Mercury* (Macrae *et al.*, 2008 ▶); software used to prepare material for publication: *SHELXL97*.

## Supplementary Material

Crystal structure: contains datablock(s) global, I. DOI: 10.1107/S1600536813013500/pk2482sup1.cif

Structure factors: contains datablock(s) I. DOI: 10.1107/S1600536813013500/pk2482Isup2.hkl

Supplementary material file. DOI: 10.1107/S1600536813013500/pk2482Isup3.cml

Additional supplementary materials:  crystallographic information; 3D view; checkCIF report

## Figures and Tables

**Table 1:** Hydrogen-bond geometry (Å, °) *Cg*1, *Cg*2, *Cg*3 and *Cg*4 are centroids of the C10–C15, C30–C35, N1/N2/C1–C4 and N21/N22/C21–C24 rings, respectively.

*D*—H⋯*A*	*D*—H	H⋯*A*	*D*⋯*A*	*D*—H⋯*A*
N5—H5*N*⋯N23	0.896 (13)	2.129 (11)	2.9883 (13)	160.2 (12)
N25—H25*N*⋯N3	0.908 (12)	2.151 (12)	3.0088 (15)	157.2 (12)
C25—H25⋯*Cg*1	0.95	2.76	3.6413 (14)	156
C5—H5⋯*Cg*2	0.95	2.72	3.6179 (13)	158
C12—H12⋯*Cg*3^i^	0.95	2.93	3.6703 (17)	135
C15—H15⋯*Cg*4^ii^	0.95	2.60	3.5158 (15)	161

Symmetry codes: (i) 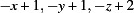; (ii) 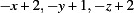.

**Fig. 1.**
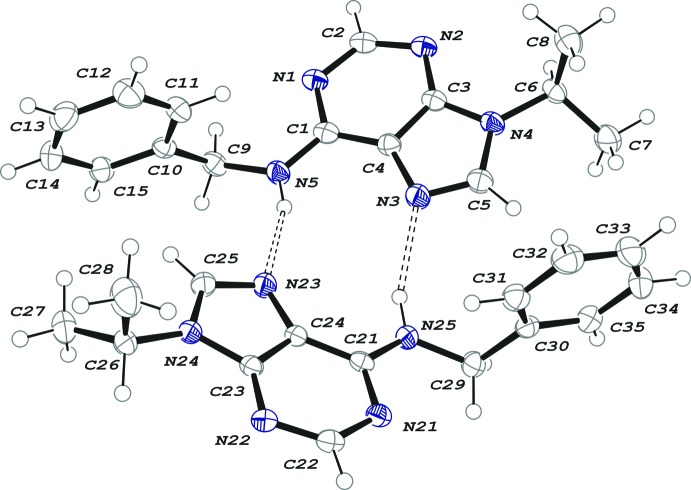
An ellipsoid plot of the asymmetric unit with atoms represented as 50% probability ellipsoids. H-atoms are showed as small spheres of arbitrary radii. H-bonds are denoted with dashed lines.

**Fig. 2.**
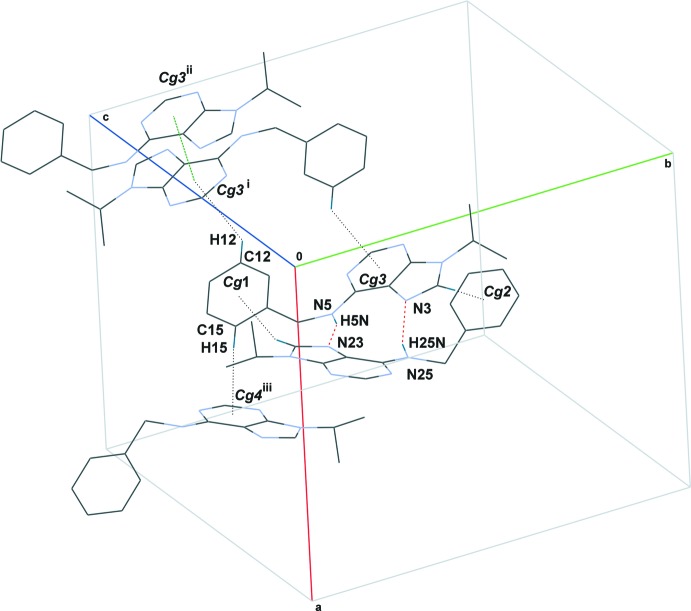
A fragment of the molecular packing. H-atoms have been omitted for clarity with exception to those participating in weak interactions. The red dashed lines denote the H-bonds, the green dashed lines denote the π···π interactions and the black dotted lines denote the C—H···π contacts. Cg1, Cg2,Cg3 and Cg4 are respective centers of gravity of C10–C15; C30–C35; C1,N1,C2,N2,C3,C4 and C21,N21,C22,N22,C23,C24 rings. Symmetry codes: i) -x + 1, -y + 1, -z + 2; ii) x, y, z + 1; iii) -x + 2, -y + 1, -z + 2.

## supplementary crystallographic information

### Comment

The synthesis of purine derivatives bearing various substituents at C6 and N9 positions is closely related with the eventual biological activity of the final molecule. Recently, several 6,9-disubstituted purines were described as antibacterial (Tunçbilek *et al.*, 2009), antileishmanial (Jorda *et al.*, 2011) and antitumor (Cappellacci *et al.*, 2011) agents. The title molecule, *N*-benzyl-9-isopropyl-9*H*-purin-6-amine, was prepared as a part of our ongoing research aimed at preparation of new disubstituted purine series.

The asymmetric unit of the title compound consists of two purine-based molecules slightly different in their geometries (Fig. 1). The dihedral angles between the best planes of the purine and benzene rings are 89.21 (3)° and 82.14 (4)°, respectively. The torsion angles N1—C1—N5—C9, C1—N5—C9—C10, N5—C9—C10—C11 and C5—N4—C6—C7 indicating the mutual orientation of substituents and purine ring are 2.85 (19), -110.7 (14), 33.8 (18) and 31.3 (18)°, respectively. The corresponding values of torsion angles for the second conformer are 0.44 (19), -96.7 (15), 35.5 (17) and 35.9 (18)°, respectively. The molecules within the asymmetric unit are linked by N5—H5N···N23 and N25—H25N···N3 hydrogen bonds (Table 1, Fig. 2) to form dimers with dihedral angles between the best planes of the two purines being 32.53 (3)°. In contrast, the benzene rings are essentially parallel with the dihedral angle of 2.88 (3)°. The crystal packing is further stabilized *via* short intermolecular non-bonding C—H···π contacts and π···π interactions (Table 1, Fig. 2). The interplanar distance between the adjacent purine best planes of the π···π stacked molecules is of 3.2583 (13) Å.

### Experimental

The title compound was prepared according to a slightly modified literature procedure (Fiorini & Abel, 1998). 6-Chloro-9-isopropyl-9*H*-purine (100 mg, 0.51 mmol) and benzylamine (58 mg, 0.54 mmol) were dissolved in a mixture of DMSO (4 ml) and triethylamine (57 mg, 0.56 mmol). The resulting solution was stirred at 90 °C for 2 h. Subsequently, the mixture was diluted with water and extracted with diethyl ether (7 × 10 ml). The combined organic layers were washed twice with brine, dried over Na_2_SO_4_ and evaporated in vacuum. The desired product was obtained after the purification of crude material using column chromatography (silica gel; petroleum ether/ethyl acetate, 1/3, *v*/*v*) as a colourless crystalline powder (66 mg, 49%, mp 388–391 K). The crystals used for data collection were grown by spontaneous evaporation from deuterochloroform at room temperature.

### Refinement

All carbon bound H atoms were placed at calculated positions and were refined as riding with their *U*_iso_ set to either 1.2*U*_eq_ or 1.5*U*_eq_ (methyl) of the respective carrier atoms; in addition, the methyl H atoms were allowed to rotate about the C—C bond. Nitrogen bound H atoms were located in a difference Fourier map and refined isotropically.

### Crystal data

**Table:**

C_15_H_17_N_5_	*F*(000) = 1136
*M**_r_* = 267.34	*D*_x_ = 1.258 Mg m^−^^3^
Monoclinic, *P*2_1_/*c*	Melting point: 390 K
Hall symbol: -P 2ybc	Mo *K*α radiation, λ = 0.71073 Å
*a* = 12.9926 (5) Å	Cell parameters from 6488 reflections
*b* = 21.1673 (7) Å	θ = 2.9–27.3°
*c* = 11.2622 (6) Å	µ = 0.08 mm^−^^1^
β = 114.274 (5)°	*T* = 120 K
*V* = 2823.5 (2) Å^3^	Pyramid, colourless
*Z* = 8	0.50 × 0.38 × 0.20 mm

### Data collection

**Table:**

Oxford Diffraction Xcalibur (Sapphire2) diffractometer	4972 independent reflections
Radiation source: Enhance (Mo) X-ray Source	3280 reflections with *I* > 2σ(*I*)
Graphite monochromator	*R*_int_ = 0.035
Detector resolution: 8.4353 pixels mm^-1^	θ_max_ = 25.0°, θ_min_ = 3.3°
ω scan	*h* = −15→15
Absorption correction: multi-scan (*CrysAlis RED*; Oxford Diffraction, 2009)	*k* = −25→23
*T*_min_ = 0.942, *T*_max_ = 1.000	*l* = −13→10
21460 measured reflections	

### Refinement

**Table:**

Refinement on *F*^2^	0 restraints
Least-squares matrix: full	H atoms treated by a mixture of independent and constrained refinement
*R*[*F*^2^ > 2σ(*F*^2^)] = 0.029	*w* = 1/[σ^2^(*F*_o_^2^) + (0.0275*P*)^2^] where *P* = (*F*_o_^2^ + 2*F*_c_^2^)/3
*wR*(*F*^2^) = 0.058	(Δ/σ)_max_ = 0.001
*S* = 0.83	Δρ_max_ = 0.13 e Å^−^^3^
4972 reflections	Δρ_min_ = −0.16 e Å^−^^3^
373 parameters	

### Special details

**Table:**

Geometry. All e.s.d.'s (except the e.s.d. in the dihedral angle between two l.s. planes) are estimated using the full covariance matrix. The cell e.s.d.'s are taken into account individually in the estimation of e.s.d.'s in distances, angles and torsion angles; correlations between e.s.d.'s in cell parameters are only used when they are defined by crystal symmetry. An approximate (isotropic) treatment of cell e.s.d.'s is used for estimating e.s.d.'s involving l.s. planes.
Refinement. Refinement of *F*^2^ against ALL reflections. The weighted *R*-factor *wR* and goodness of fit *S* are based on *F*^2^, conventional *R*-factors *R* are based on *F*, with *F* set to zero for negative *F*^2^. The threshold expression of *F*^2^ > 2σ(*F*^2^) is used only for calculating *R*-factors(gt) *etc*. and is not relevant to the choice of reflections for refinement. *R*-factors based on *F*^2^ are statistically about twice as large as those based on *F*, and *R*- factors based on ALL data will be even larger.

### Fractional atomic coordinates and isotropic or equivalent isotropic displacement parameters (Å^2^)

**Table:**

	*x*	*y*	*z*	*U*_iso_*/*U*_eq_	
N1	0.48434 (8)	0.47471 (5)	0.65985 (10)	0.0260 (3)	
N2	0.35401 (8)	0.55081 (5)	0.51464 (10)	0.0262 (3)	
N3	0.61994 (8)	0.62964 (5)	0.66899 (10)	0.0313 (3)	
N4	0.44524 (8)	0.65164 (5)	0.51961 (10)	0.0263 (3)	
N5	0.67119 (9)	0.49857 (5)	0.79188 (10)	0.0265 (3)	
C1	0.57003 (10)	0.51658 (6)	0.70266 (12)	0.0241 (3)	
C2	0.38440 (10)	0.49452 (6)	0.57007 (12)	0.0279 (3)	
H2	0.3265	0.4634	0.5419	0.033*	
C3	0.44264 (10)	0.59076 (6)	0.55935 (12)	0.0235 (3)	
C4	0.54997 (10)	0.57782 (6)	0.65076 (11)	0.0235 (3)	
C5	0.55292 (10)	0.67225 (6)	0.58899 (12)	0.0324 (3)	
H5	0.5775	0.7136	0.5804	0.039*	
C6	0.34853 (10)	0.68646 (6)	0.42406 (12)	0.0264 (3)	
H6	0.2996	0.6553	0.3584	0.032*	
C7	0.38698 (11)	0.73471 (7)	0.35260 (13)	0.0440 (4)	
H7A	0.4339	0.7141	0.3146	0.066*	
H7B	0.4310	0.7676	0.4136	0.066*	
H7C	0.3210	0.7538	0.2831	0.066*	
C8	0.27902 (11)	0.71513 (6)	0.48996 (13)	0.0374 (4)	
H8A	0.2602	0.6824	0.5394	0.056*	
H8B	0.2093	0.7329	0.4239	0.056*	
H8C	0.3224	0.7487	0.5493	0.056*	
C9	0.69743 (10)	0.43519 (6)	0.84296 (12)	0.0275 (3)	
H9A	0.7664	0.4207	0.8340	0.033*	
H9B	0.6349	0.4068	0.7898	0.033*	
C10	0.71588 (10)	0.42924 (6)	0.98378 (12)	0.0226 (3)	
C11	0.65661 (10)	0.46597 (6)	1.03600 (13)	0.0289 (3)	
H11	0.6031	0.4958	0.9824	0.035*	
C12	0.67436 (11)	0.45977 (7)	1.16490 (13)	0.0369 (4)	
H12	0.6325	0.4850	1.1992	0.044*	
C13	0.75233 (11)	0.41715 (7)	1.24409 (14)	0.0381 (4)	
H13	0.7646	0.4131	1.3329	0.046*	
C14	0.81238 (11)	0.38046 (6)	1.19388 (14)	0.0363 (4)	
H14	0.8666	0.3511	1.2481	0.044*	
C15	0.79357 (10)	0.38652 (6)	1.06441 (13)	0.0289 (3)	
H15	0.8349	0.3608	1.0302	0.035*	
N21	1.00117 (8)	0.72864 (5)	0.95195 (10)	0.0239 (3)	
N22	1.08432 (8)	0.68113 (5)	1.16590 (9)	0.0236 (3)	
N23	0.87221 (8)	0.57374 (5)	0.96305 (10)	0.0279 (3)	
N24	0.99893 (8)	0.57966 (5)	1.17200 (10)	0.0256 (3)	
N25	0.85603 (9)	0.68204 (5)	0.77541 (10)	0.0260 (3)	
C21	0.92837 (9)	0.68029 (6)	0.90160 (12)	0.0220 (3)	
C22	1.07258 (10)	0.72557 (6)	1.07788 (12)	0.0252 (3)	
H22	1.1224	0.7605	1.1090	0.030*	
C23	1.01033 (9)	0.63394 (6)	1.11174 (12)	0.0214 (3)	
C24	0.93237 (9)	0.62980 (6)	0.98385 (12)	0.0215 (3)	
C25	0.91468 (10)	0.54632 (6)	1.07785 (13)	0.0310 (3)	
H25	0.8889	0.5066	1.0940	0.037*	
C26	1.05832 (10)	0.56472 (6)	1.31163 (12)	0.0297 (3)	
H26	1.1349	0.5846	1.3442	0.036*	
C27	1.07405 (11)	0.49430 (6)	1.33211 (13)	0.0417 (4)	
H27A	1.1117	0.4776	1.2791	0.063*	
H27B	1.0002	0.4740	1.3062	0.063*	
H27C	1.1204	0.4856	1.4243	0.063*	
C28	0.99545 (12)	0.59390 (7)	1.38523 (14)	0.0486 (4)	
H28A	0.9907	0.6397	1.3718	0.073*	
H28B	1.0358	0.5847	1.4783	0.073*	
H28C	0.9192	0.5761	1.3532	0.073*	
C29	0.85268 (10)	0.73381 (6)	0.68906 (12)	0.0278 (3)	
H29A	0.8387	0.7163	0.6023	0.033*	
H29B	0.9275	0.7546	0.7232	0.033*	
C30	0.76355 (10)	0.78312 (6)	0.67322 (12)	0.0246 (3)	
C31	0.73658 (10)	0.79940 (6)	0.77588 (13)	0.0307 (3)	
H31	0.7761	0.7802	0.8586	0.037*	
C32	0.65295 (11)	0.84318 (6)	0.75992 (14)	0.0376 (4)	
H32	0.6350	0.8537	0.8312	0.045*	
C33	0.59581 (12)	0.87150 (6)	0.64080 (16)	0.0429 (4)	
H33	0.5379	0.9013	0.6295	0.051*	
C34	0.62262 (11)	0.85664 (6)	0.53779 (15)	0.0405 (4)	
H34	0.5840	0.8766	0.4558	0.049*	
C35	0.70600 (10)	0.81253 (6)	0.55402 (13)	0.0321 (3)	
H35	0.7240	0.8023	0.4827	0.038*	
H5N	0.7264 (11)	0.5272 (6)	0.8262 (12)	0.037 (4)*	
H25N	0.7954 (10)	0.6558 (6)	0.7491 (12)	0.037 (4)*	

### Atomic displacement parameters (Å^2^)

**Table:**

	*U*^11^	*U*^22^	*U*^33^	*U*^12^	*U*^13^	*U*^23^
N1	0.0217 (6)	0.0304 (6)	0.0255 (6)	−0.0021 (5)	0.0093 (5)	−0.0038 (5)
N2	0.0209 (6)	0.0308 (7)	0.0259 (7)	−0.0027 (5)	0.0087 (5)	−0.0041 (5)
N3	0.0241 (6)	0.0316 (7)	0.0299 (7)	−0.0035 (5)	0.0026 (5)	0.0040 (6)
N4	0.0201 (6)	0.0298 (7)	0.0243 (6)	0.0000 (5)	0.0043 (5)	0.0002 (5)
N5	0.0222 (6)	0.0272 (7)	0.0248 (7)	−0.0012 (5)	0.0045 (5)	0.0000 (5)
C1	0.0220 (7)	0.0327 (8)	0.0194 (8)	−0.0008 (6)	0.0103 (6)	−0.0048 (6)
C2	0.0223 (7)	0.0328 (8)	0.0292 (8)	−0.0045 (6)	0.0114 (6)	−0.0062 (7)
C3	0.0206 (7)	0.0300 (8)	0.0204 (8)	−0.0003 (6)	0.0090 (6)	−0.0034 (6)
C4	0.0213 (7)	0.0280 (8)	0.0198 (8)	−0.0021 (6)	0.0072 (6)	−0.0017 (6)
C5	0.0236 (7)	0.0333 (8)	0.0328 (9)	−0.0048 (6)	0.0042 (6)	0.0021 (7)
C6	0.0214 (7)	0.0328 (8)	0.0201 (8)	0.0026 (6)	0.0036 (6)	−0.0001 (6)
C7	0.0321 (8)	0.0602 (11)	0.0350 (9)	0.0074 (7)	0.0092 (7)	0.0188 (8)
C8	0.0387 (8)	0.0418 (9)	0.0317 (9)	0.0115 (7)	0.0144 (7)	0.0023 (7)
C9	0.0248 (7)	0.0263 (8)	0.0299 (8)	0.0037 (6)	0.0097 (6)	−0.0034 (6)
C10	0.0199 (7)	0.0197 (7)	0.0274 (8)	−0.0027 (6)	0.0089 (6)	−0.0033 (6)
C11	0.0247 (7)	0.0306 (8)	0.0298 (9)	0.0048 (6)	0.0097 (6)	0.0000 (7)
C12	0.0360 (8)	0.0450 (10)	0.0338 (9)	0.0042 (7)	0.0186 (7)	−0.0038 (7)
C13	0.0426 (9)	0.0442 (9)	0.0302 (9)	−0.0042 (7)	0.0176 (7)	0.0040 (7)
C14	0.0370 (8)	0.0307 (9)	0.0387 (10)	0.0045 (7)	0.0132 (7)	0.0126 (7)
C15	0.0289 (7)	0.0216 (8)	0.0379 (9)	0.0034 (6)	0.0154 (7)	0.0014 (6)
N21	0.0202 (6)	0.0243 (6)	0.0270 (7)	0.0002 (5)	0.0094 (5)	−0.0011 (5)
N22	0.0202 (5)	0.0251 (6)	0.0251 (6)	−0.0022 (5)	0.0090 (5)	−0.0015 (5)
N23	0.0248 (6)	0.0261 (6)	0.0273 (7)	−0.0031 (5)	0.0050 (5)	0.0033 (5)
N24	0.0201 (6)	0.0278 (6)	0.0231 (6)	−0.0041 (5)	0.0029 (5)	0.0037 (5)
N25	0.0223 (6)	0.0270 (7)	0.0245 (7)	−0.0016 (5)	0.0054 (5)	0.0042 (5)
C21	0.0164 (6)	0.0249 (7)	0.0256 (8)	0.0030 (6)	0.0097 (6)	−0.0019 (6)
C22	0.0206 (7)	0.0256 (8)	0.0301 (8)	−0.0015 (6)	0.0112 (6)	−0.0043 (7)
C23	0.0168 (6)	0.0228 (7)	0.0246 (8)	0.0011 (5)	0.0086 (6)	0.0005 (6)
C24	0.0173 (6)	0.0224 (7)	0.0235 (8)	0.0001 (5)	0.0071 (6)	0.0006 (6)
C25	0.0251 (7)	0.0281 (8)	0.0323 (9)	−0.0071 (6)	0.0043 (7)	0.0045 (7)
C26	0.0228 (7)	0.0369 (9)	0.0226 (8)	−0.0038 (6)	0.0023 (6)	0.0069 (6)
C27	0.0379 (8)	0.0416 (9)	0.0370 (9)	0.0032 (7)	0.0065 (7)	0.0145 (7)
C28	0.0530 (10)	0.0584 (11)	0.0321 (9)	0.0091 (8)	0.0151 (8)	0.0064 (8)
C29	0.0286 (7)	0.0310 (8)	0.0243 (8)	0.0016 (6)	0.0114 (6)	0.0043 (6)
C30	0.0228 (7)	0.0248 (8)	0.0260 (8)	−0.0036 (6)	0.0099 (6)	0.0011 (6)
C31	0.0299 (7)	0.0313 (8)	0.0298 (8)	−0.0023 (6)	0.0111 (7)	0.0000 (7)
C32	0.0374 (8)	0.0327 (9)	0.0488 (10)	0.0000 (7)	0.0238 (8)	−0.0045 (8)
C33	0.0343 (8)	0.0288 (9)	0.0654 (12)	0.0067 (7)	0.0203 (8)	0.0028 (8)
C34	0.0380 (9)	0.0313 (9)	0.0468 (10)	0.0057 (7)	0.0119 (8)	0.0120 (8)
C35	0.0325 (8)	0.0315 (8)	0.0324 (9)	−0.0007 (6)	0.0136 (7)	0.0053 (7)

### Geometric parameters (Å, º)

**Table:**

N1—C2	1.3434 (15)	N21—C22	1.3396 (14)
N1—C1	1.3476 (15)	N21—C21	1.3500 (14)
N2—C2	1.3276 (15)	N22—C22	1.3291 (14)
N2—C3	1.3482 (14)	N22—C23	1.3460 (14)
N3—C5	1.3185 (15)	N23—C25	1.3138 (14)
N3—C4	1.3851 (14)	N23—C24	1.3869 (14)
N4—C5	1.3635 (14)	N24—C25	1.3661 (14)
N4—C3	1.3693 (15)	N24—C23	1.3730 (14)
N4—C6	1.4725 (14)	N24—C26	1.4733 (14)
N5—C1	1.3404 (15)	N25—C21	1.3440 (15)
N5—C9	1.4441 (14)	N25—C29	1.4539 (15)
N5—H5N	0.897 (12)	N25—H25N	0.908 (12)
C1—C4	1.4017 (16)	C21—C24	1.4012 (16)
C2—H2	0.9500	C22—H22	0.9500
C3—C4	1.3781 (15)	C23—C24	1.3815 (15)
C5—H5	0.9500	C25—H25	0.9500
C6—C7	1.5069 (17)	C26—C27	1.5093 (17)
C6—C8	1.5119 (16)	C26—C28	1.5141 (18)
C6—H6	1.0000	C26—H26	1.0000
C7—H7A	0.9800	C27—H27A	0.9800
C7—H7B	0.9800	C27—H27B	0.9800
C7—H7C	0.9800	C27—H27C	0.9800
C8—H8A	0.9800	C28—H28A	0.9800
C8—H8B	0.9800	C28—H28B	0.9800
C8—H8C	0.9800	C28—H28C	0.9800
C9—C10	1.5086 (16)	C29—C30	1.5141 (16)
C9—H9A	0.9900	C29—H29A	0.9900
C9—H9B	0.9900	C29—H29B	0.9900
C10—C15	1.3811 (16)	C30—C31	1.3826 (17)
C10—C11	1.3842 (16)	C30—C35	1.3869 (16)
C11—C12	1.3794 (17)	C31—C32	1.3825 (17)
C11—H11	0.9500	C31—H31	0.9500
C12—C13	1.3757 (17)	C32—C33	1.3753 (18)
C12—H12	0.9500	C32—H32	0.9500
C13—C14	1.3764 (18)	C33—C34	1.3778 (19)
C13—H13	0.9500	C33—H33	0.9500
C14—C15	1.3818 (17)	C34—C35	1.3841 (17)
C14—H14	0.9500	C34—H34	0.9500
C15—H15	0.9500	C35—H35	0.9500
			
C2—N1—C1	117.70 (11)	C22—N21—C21	117.94 (11)
C2—N2—C3	110.12 (10)	C22—N22—C23	110.22 (10)
C5—N3—C4	103.39 (10)	C25—N23—C24	103.43 (10)
C5—N4—C3	105.74 (10)	C25—N24—C23	105.28 (10)
C5—N4—C6	128.68 (11)	C25—N24—C26	128.21 (11)
C3—N4—C6	125.56 (10)	C23—N24—C26	126.27 (10)
C1—N5—C9	124.12 (11)	C21—N25—C29	122.94 (11)
C1—N5—H5N	119.7 (8)	C21—N25—H25N	117.6 (8)
C9—N5—H5N	116.1 (8)	C29—N25—H25N	117.3 (8)
N5—C1—N1	119.51 (12)	N25—C21—N21	119.20 (12)
N5—C1—C4	122.18 (11)	N25—C21—C24	122.67 (11)
N1—C1—C4	118.31 (11)	N21—C21—C24	118.13 (11)
N2—C2—N1	129.98 (12)	N22—C22—N21	129.88 (11)
N2—C2—H2	115.0	N22—C22—H22	115.1
N1—C2—H2	115.0	N21—C22—H22	115.1
N2—C3—N4	126.89 (11)	N22—C23—N24	126.80 (11)
N2—C3—C4	126.94 (12)	N22—C23—C24	126.90 (12)
N4—C3—C4	106.13 (11)	N24—C23—C24	106.28 (10)
C3—C4—N3	110.75 (11)	C23—C24—N23	110.56 (11)
C3—C4—C1	116.93 (11)	C23—C24—C21	116.92 (11)
N3—C4—C1	132.26 (11)	N23—C24—C21	132.52 (11)
N3—C5—N4	113.99 (12)	N23—C25—N24	114.45 (11)
N3—C5—H5	123.0	N23—C25—H25	122.8
N4—C5—H5	123.0	N24—C25—H25	122.8
N4—C6—C7	111.03 (10)	N24—C26—C27	110.72 (11)
N4—C6—C8	110.17 (10)	N24—C26—C28	109.45 (11)
C7—C6—C8	112.72 (11)	C27—C26—C28	112.88 (12)
N4—C6—H6	107.6	N24—C26—H26	107.9
C7—C6—H6	107.6	C27—C26—H26	107.9
C8—C6—H6	107.6	C28—C26—H26	107.9
C6—C7—H7A	109.5	C26—C27—H27A	109.5
C6—C7—H7B	109.5	C26—C27—H27B	109.5
H7A—C7—H7B	109.5	H27A—C27—H27B	109.5
C6—C7—H7C	109.5	C26—C27—H27C	109.5
H7A—C7—H7C	109.5	H27A—C27—H27C	109.5
H7B—C7—H7C	109.5	H27B—C27—H27C	109.5
C6—C8—H8A	109.5	C26—C28—H28A	109.5
C6—C8—H8B	109.5	C26—C28—H28B	109.5
H8A—C8—H8B	109.5	H28A—C28—H28B	109.5
C6—C8—H8C	109.5	C26—C28—H28C	109.5
H8A—C8—H8C	109.5	H28A—C28—H28C	109.5
H8B—C8—H8C	109.5	H28B—C28—H28C	109.5
N5—C9—C10	113.94 (10)	N25—C29—C30	114.18 (11)
N5—C9—H9A	108.8	N25—C29—H29A	108.7
C10—C9—H9A	108.8	C30—C29—H29A	108.7
N5—C9—H9B	108.8	N25—C29—H29B	108.7
C10—C9—H9B	108.8	C30—C29—H29B	108.7
H9A—C9—H9B	107.7	H29A—C29—H29B	107.6
C15—C10—C11	118.18 (12)	C31—C30—C35	118.45 (12)
C15—C10—C9	120.21 (11)	C31—C30—C29	121.18 (11)
C11—C10—C9	121.60 (11)	C35—C30—C29	120.36 (12)
C12—C11—C10	120.81 (12)	C32—C31—C30	120.95 (13)
C12—C11—H11	119.6	C32—C31—H31	119.5
C10—C11—H11	119.6	C30—C31—H31	119.5
C13—C12—C11	120.31 (13)	C33—C32—C31	119.95 (14)
C13—C12—H12	119.8	C33—C32—H32	120.0
C11—C12—H12	119.8	C31—C32—H32	120.0
C12—C13—C14	119.63 (13)	C32—C33—C34	119.99 (13)
C12—C13—H13	120.2	C32—C33—H33	120.0
C14—C13—H13	120.2	C34—C33—H33	120.0
C13—C14—C15	119.79 (13)	C33—C34—C35	119.86 (14)
C13—C14—H14	120.1	C33—C34—H34	120.1
C15—C14—H14	120.1	C35—C34—H34	120.1
C10—C15—C14	121.27 (12)	C34—C35—C30	120.79 (13)
C10—C15—H15	119.4	C34—C35—H35	119.6
C14—C15—H15	119.4	C30—C35—H35	119.6

### Hydrogen-bond geometry (Å, º)

*Cg*1, *Cg*2, *Cg*3 and *Cg*4 are centroids of the C10–C15, C30–C35, N1/N2/C1–C4 and N21/N22/C21–C24 rings, respectively.

**Table:**

*D*—H···*A*	*D*—H	H···*A*	*D*···*A*	*D*—H···*A*
N5—H5*N*···N23	0.896 (13)	2.129 (11)	2.9883 (13)	160.2 (12)
N25—H25*N*···N3	0.908 (12)	2.151 (12)	3.0088 (15)	157.2 (12)
C25—H25···*Cg*1	0.95	2.76	3.6413 (14)	156
C5—H5···*Cg*2	0.95	2.72	3.6179 (13)	158
C12—H12···*Cg*3^i^	0.95	2.93	3.6703 (17)	135
C15—H15···*Cg*4^ii^	0.95	2.60	3.5158 (15)	161
*Cg*3···*Cg*3^iii^			3.3071 (1)	

Symmetry codes: (i) −*x*+1, −*y*+1, −*z*+2; (ii) −*x*+2, −*y*+1, −*z*+2; (iii) −*x*+1, −*y*+1, −*z*+1.
